# Decreased expression of bone morphogenetic protein-2 is correlated with biochemical recurrence in prostate cancer: Immunohistochemical analysis

**DOI:** 10.1038/s41598-018-28566-9

**Published:** 2018-07-16

**Authors:** Bum Sik Tae, Seok Cho, Hyun Cheol Kim, Cheol Hwan Kim, Seok Ho Kang, Jeong Gu Lee, Je Jong Kim, Hong Seok Park, Jun Cheon, Mi Mi Oh, Sung Gu Kang

**Affiliations:** 1 Department of Urology, Korea University College of Medicine, Seoul, Korea; 2 Department of Urology, Ilsan Paik Hospital, Inje University College of Medicine, Goyang, Korea; 3 Department of Pathology, Hallym University Dongtan Sacred Heart Hospital, Hwaseong, Republic of Korea; 4 Department of Pathology, Korea University College of Medicine, Seoul, Korea

## Abstract

We evaluated the prognostic value of BMP-2 expression in prostate cancer tissue via immunohistochemistry in prostate cancer patients. From July 2007 to August 2010, radical prostatectomy specimens from 90 patients with clinically localized prostate cancer (mean age, 62.7 years, mean follow-up 90.4 months) were assessed for BMP-2 expression using immunohistochemistry. We used stepwise multivariate Cox regression models stratified by study to assess the independent effects of the predictive factors and estimated hazard ratios (HRs). There were significant differences in the baseline characteristics of Gleason score (GS) and biochemical recurrence (BCR) between the groups with decreased and normal BMP-2 expression. Univariate analysis revealed GS, T stage (≥T3), and decreased BMP-2 expression as significant predictive determinants of BCR. In addition, GS (7: HR 2.836, p = 0.022; ≥8: HR 3.506, p = 0.048) and decreased BMP-2 expression (HR 2.007, p = 0.047) were significantly correlated with BCR in multivariate analysis. Overall five-year BCR-free survival rates in the group with decreased BMP-2 expression were worse than those in the group with normal expression. Therefore, decreased BMP-2 expression in prostate cancer tissue was correlated with the prognostic factors for BCR-free survival in patients with prostate cancer.

## Introduction

Prostate cancer (PCa) is the most commonly diagnosed non-cutaneous malignancy in men and is the second leading cause of cancer-related death in men in the United States^1^. The natural history of PCa varies from an indolent tumour to a highly aggressive cancer^2^. Although numerous studies have been performed on PCa, there have been few reliable studies regarding predictive markers for PCa progression. The socioeconomic burden of PCa treatment continues to increase, negatively impacting the health and quality of life of patients. Hence, it is critical to identify new, reliable prognostic markers able to differentiate between indolent and aggressive PCa tumours. Excessive treatment can potentially be avoided once an adequate marker or sets of markers to differentiate an indolent tumour from a highly aggressive cancer are made available.

Bone morphogenetic proteins (BMPs), members of the transforming growth factor beta (TGF-β) superfamily, are multifunctional cytokines^3^. BMPs were originally described as osteoinductive cytokines that stimulate the formation of bone and cartilage^4^. Subsequently, BMPs have been shown to play important roles during development and in the regulation of various cellular processes, such as migration, proliferation, apoptosis, and differentiation of numerous cell types.

In addition to the physiological roles of BMPs, recent studies have revealed that BMPs play a potential role in promoting primary tumorigenesis and facilitating tumour metastasis^5^. However, the results of these studies are often contradictory. As the same ligand can act differently depending on the cancer type, it may be necessary for multiple members of the BMP family to be examined independently^6^. Specific isoforms of BMPs have shown oncogenic properties whereas others have demonstrated tumour suppressor activity. The current consensus is that BMPs are involved in both the promotion and inhibition of cancer progression depending on the type of cancer cells and the specific isoforms of BMPs.

BMP-2 plays a role in osteoblast differentiation, osteogenesis, and chondrogenesis. BMP-2 also has potential roles in apoptosis and as a retinoid mediator^5^. In previous studies, BMP-2 was shown to promote tumorigenesis and metastatic features in a PCa cell line; however, other studies showed that BMP-2 did not promote tumorigenesis^7–10^. Consequently, the biological effects of BMP-2 on PCa development and progression remain unclear, as only limited information is available regarding the role of BMP-2 in human PCa.

The European Association of Urology guidelines state that radical prostatectomy (RP) is the only surgical treatment option for localised prostate cancer^11^. However, it is known that approximately 15–30% of RP-treated patients experience biochemical recurrence (BCR) within five years and 40% within 10 years^12^. Previous reports presented that post-RP BCR is correlated with initial serum prostate-specific antigen (PSA) level, Gleason score (GS), post-operative surgical margins (PSM), and pathological stage^11^. However, the predictive accuracy of BCR was not yet satisfactory, and the time to post-RP BCR is the most important predictor of PCa-specific mortality^13^. Therefore, identification of high-risk patients for post-RP BCR will enable early adjuvant treatment for those patients, reducing the risk for disease progression and PCa-specific mortality^14^. In the present study, we examined the expression level of BMP-2 in cancerous prostate tissues via immunohistochemistry and investigated its prognostic significance in predicting post-RP BCR in patients with localised PCa.

## Materials and Methods

### Ethic statement

This study was approved by the ethics board of the Korea University Anam Hospital (IRB No. 2011AN0228), and all patients provided written informed consent. Patient information was anonymised and de-identified prior to the study, and we carried out all study procedures in accordance with the Declaration of Helsinki guidelines. All methods in this study were performed in accordance with the relevant guidelines and regulations.

### Patients and Definition

This research was designed as a retrospective study. Data were gathered from 137 consecutive patients who underwent RP at Korea University Hospital between 2007 and 2010. Among these patients, 27 who received preoperative neoadjuvant therapy and two lost at follow-up were excluded from the analysis. Therefore, the clinicopathologic parameters of 90 patients were analysed in this study, including age, initial PSA, tumour grade according to the GS, tumour stage (T stage), PSM from specimens, follow-up duration after prostatectomy, and PSA levels during follow-up. Follow-up examinations were performed every three months for the first two years after surgery, every six months over the following three years, and on an annual basis thereafter. BCR after therapy was defined as two consecutive serum PSA concentration measurements >0.2 ng/mL, while clinical progression was defined as local recurrence or distant metastasis observed on imaging studies.

### Immunohistochemical staining and assessment

Immunostaining was initially assessed by one investigator (H.C.K) and subsequently reviewed independently by a histopathologist (C.H.K), both of whom were blinded to the patient clinical courses. Using the technique of Kononen and researchers, medium-density tissue microarrays were constructed using tissue core biopsies of 1.5 or 2 mm^15^. For immunohistochemistry, tissue slices were collected on glass slides coated with 3-aminopropyltriethoxysilane (APES, Sigma-Aldrich, St. Louis, MO, USA), air-dried, and stored at 4 °C until processing for indirect immunoperoxidase staining, as described by Bobinac and researchers^16^. Tissue slices were deparaffinised in xylene and rehydrated in ethanol. Endogenous peroxidase and nonspecific binding were blocked by incubation in 0.3% H_2_O_2_ diluted with methanol and 5% non-immune serum. The sections were incubated with the primary antibody for 60 minutes.

The anti-BMP-2 antibody purchased from Santa Cruz Biotechnology (Santa Cruz, CA, USA) was a goat polyclonal antibody raised against a peptide mapping to the amino terminus of BMP-2. After incubation with a primary antibody, the secondary biotinylated antibody was applied according to the manufacturer’s protocol (LSAB® + Kit Peroxidase, Dako, Carpinteria, CA, USA). Peroxidase-conjugated streptavidin was added, and the site of antigen binding was visualised using 3,3′-diaminobenzidine tetrahydrochloride as a chromogen. Sections were counterstained with haematoxylin. Control slides were processed either with normal serum replacing the specific primary antibodies or with the secondary antibody alone.

**Figure 1:**
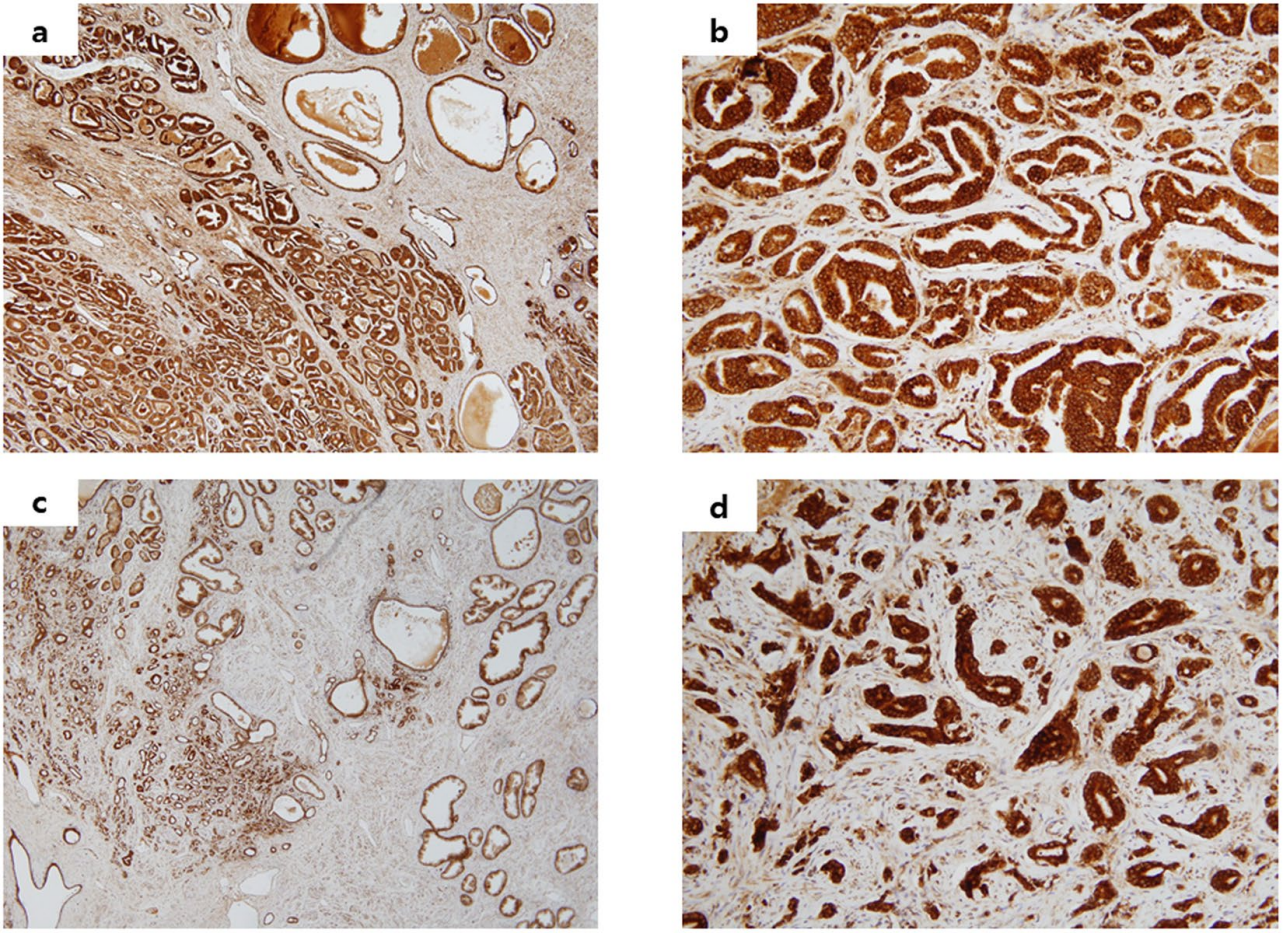
Representative photomicrographs of BMP-2 expression in malignant prostate tissue. (**a**) Normal BMP-2 expression in prostate cancer (×40). (**b**) Normal BMP-2 expression in prostate cancer (×200). (**c**) Decreased BMP-2 expression in prostate cancer (×40). (**d**) Decreased BMP-2 expression in prostate cancer (×200).

The area with the strongest positive staining in the intracellular space was selected, and the images were entered into a personal computer using a charge-coupled device colour camera (ICD-740, Ikegami, Tokyo, Japan). All images were converted to 8-bit grey-scale images consisting of 320 × 200 pixels, and their low-intensity areas were counted as the staining area using image-analysis software (NIH Image, National Institutes of Health; Bethesda, MD, USA). The proportion of low-intensity area to total-image area was calculated, and the staining rate was assessed as the mean of ten images. These investigations were conducted without knowledge of the clinical course of the patients. Cytoplasmic expression was assessed as absent (less than 10%), low (10–30%), moderate (30–50%), or high (50% or more) according to an intensity scale^17,18^. In addition, we classified expression of BMP-2 into two groups: the decreased group (absent or weak expression) and the normal group (moderate or high expression) (Fig. 1).

### Statistical analysis

Statistical analysis was performed using SPSS version 20 (SPSS Inc., Chicago, IL, USA), and all reported p-values were two-sided with p < 0.05 considered statistically significant. Chi-square tests were performed to assess individual risk factors, and factors associated with the dependent variable at a value of p < 0.05 were included in the multivariate Cox regression model. We calculated the BCR-free survival curves using the Kaplan-Meier method and compared them using the log-rank test.

## Results

### Patients and tumor characteristics

The clinical demographics of the patients are summarised in Table 1. The median age of patients was 62.7 (range: 4–74) years, and the median initial PSA level was 9.08 ng/mL (range: 3.42–37.97). GS was 6 in 28 patients (31.1%), 7 in 54 (60.0%), 8 in six (6.7%), and 9 in two (2.2%). The pathologic stage was T2a in 16 patients (17.8%), T2b in five (5.6%), T2c in 54 (60.0%), T3a in three (3.3%), and T3b in 12 (13.3%). Twenty patients were considered high-risk and were treated with immediate adjuvant hormone therapy. Four patients showed progression to castration-resistant prostate cancer (CRPC), and one died due to PCa. The mean follow-up period was 90.39 months.

**Table 1:** Clinical demographics of patients with prostate cancer (*n* = 90).

Clinical demographics		Value
Age, median (range), year		62.7 (47–74)
Initial PSA, median(range), ng/mL		9.08 (3.42–37.97)
Gleason score, n (%)		
6		28 (31.1%)
7		54 (60.0%)
8		6 (6.7%)
9		2 (2.2%)
T stage, n (%)		
T2a		16 (17.8%)
T2b		5 (5.6%)
T2c		54 (60.0%)
T3a		3 (3.3%)
T3b		12 (13.3%)
Surgical margin, n (%)		
Negative		84 (93.3%)
Positive		6 (6.7%)
Intensity of BMP-2		62.7 (47–74)
Normal expression	High expression	17 (18.9%)
	Moderate expression	41 (45.6%)
Decreased expression	Weak expression	26 (28.9%)
	Absent expression	6. (6.7%)
Immediate adjuvant hormone treatment		20 (22.2%)
Biochemical recurrence, n (%)		36 (40.0%)
Progression to CRPC, n (%)		4 (4.4%)
Follow-up, mean ± S.D months		90.39 ± 27.10

PSA  =  prostate specific antigen; BMP  =  bone morphogenetic proteins; S.D  =  standard deviation; CRPC  =  castration resistance prostate cancer.

### Prognostic value of BMP-2 in prostate cancer

Among the 90 RP-treated patients, we found BCR during the follow-up period in 36 patients (40.0%). There were significant differences in baseline characteristics of GS, pathologic stage, and BMP-2 expression between the group with BCR and the group without BCR (Table 2).

**Table 2:** Association of BCR with clinicopathological characteristics and expression of BMP-2 in 90 patients treated with radical prostatectomy for prostate cancer.

Variable	BCR group		p-value
	No BCR (N = 54)	BCR (N = 36)	
Mean age, years ± S.D	62.35 ± 6.78	63.11 ± 5.48	0.576
Mean PSA ± S.D	8.18 ± 5.74	10.43 ± 6.00	0.078
Pathological tumour stage, n (%)			0.005
≤pT2c	50 (92.6%)	25 (69.4%)	
>pT2c	4 (7.4%)	11 (30.6%)	
PSM	2 (3.7%)	4 (11.1%)	0.171
Gleason score, n (%)			0.032
6	22 (40.7%)	6 (16.7%)	
7	29 (53.7%)	25 (69.4%)	
≥8	3 (5.6%)	5 (13.9%)	
BMP-2 expression, n (%)			0.043
High	9 (16.7%)	8 (22.2%)	
Moderate	31 (57.4%)	10 (27.8%)	
Weak	11 (20.4%)	15 (41.7%)	
Absent	3 (5.6%)	3 (8.3%)	

S.D = standard deviation; BMP = bone morphogenetic proteins; PSA = prostate specific antigen; BCR = biochemical recurrence; PSM = post-operative surgical margin.

**Figure 2:**
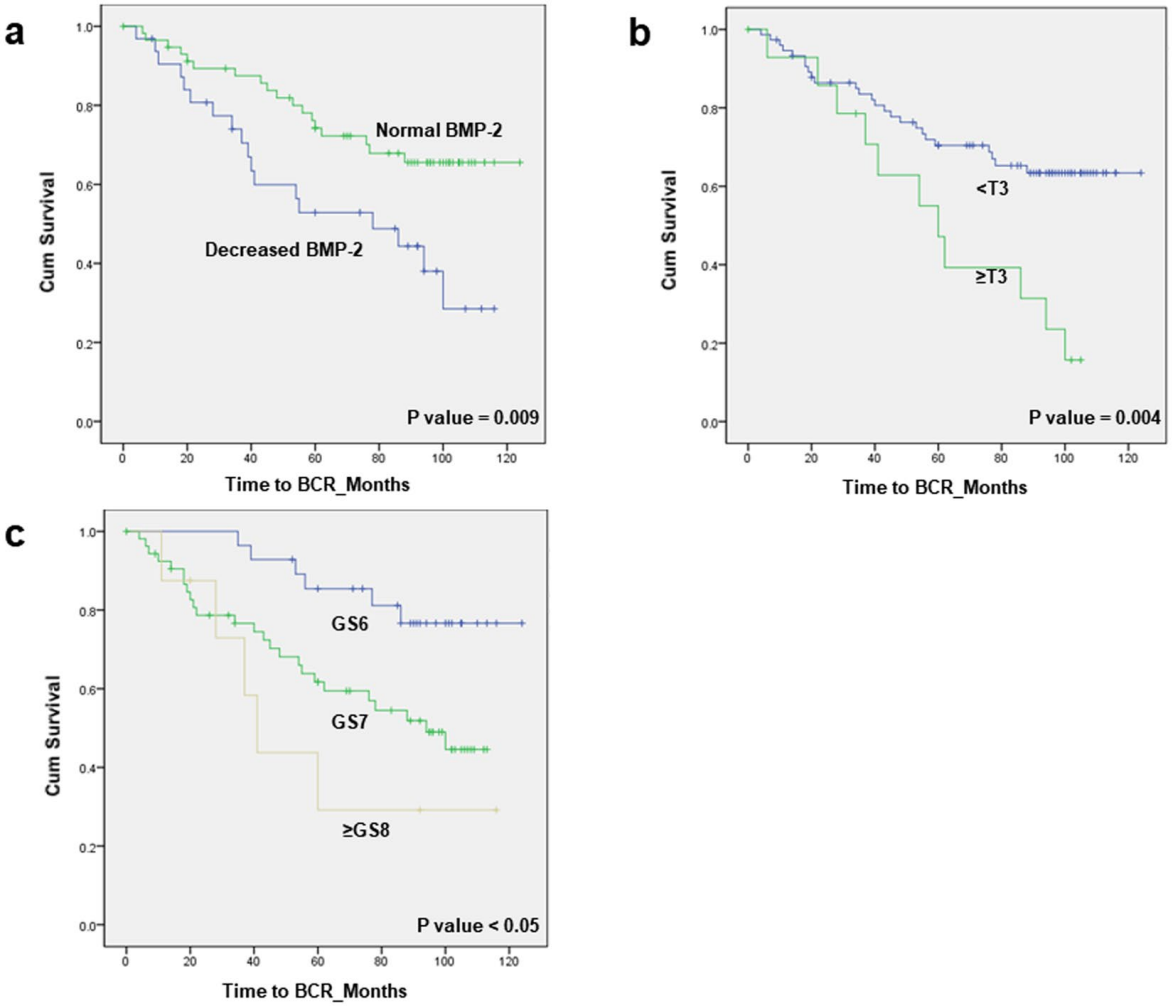
(**a**) Kaplan-Meier curves of biochemical recurrence (BCR)-free survival in patients with normal BMP expression (green) and decreased BMP expression (blue) in prostate cancer tissue. (five-year BCR-free survival, 74.3% vs. 52.9%, p = 0.009) (**b**) Kaplan-Meier curves of BCR-free survival in patients with stage ≥T3 (green) and <T3 (blue) at radical prostatectomy. (Five-year BCR-free survival, 70.4% vs. 47.1%, p = 0.004) (**c**) Kaplan-Meier curves of BCR-free survival in patients with Gleason score 6 (blue), 7 (green), and 8 (yellow) at radical prostatectomy. (Five-year BCR-free survival, 85.4% vs. 61.7% vs 29.2, p < 0.05).

Among the patients, we found decreased BMP-2 expression in 32 (36.4%). The staining rates for BMP-2 in the 90 specimens ranged from 2.0% to 76.4%. Specifically, 58 tumours were differentiated into the normal-expression staining group (mean 44.83%, range 30.2–76.4) and 32 into the low-grade group (mean 17.17%, range 2.0–29.9) (Table 3). There were significant differences in baseline characteristics of GS between the groups with normal and decreased BMP-2. A total of 36 (40.0%) patients showed BCR during follow-up, and there was a significant difference between the two groups (p = 0.018). However, no differences were observed in T stage, initial PSA, and proportion of progression to CRPC between the two groups. Univariate analysis revealed GS (7, hazard ratio [HR] 3.000, p = 0.016; ≥8, HR 5.081, p = 0.008), >T2c stage (HR 2.718, p = 0.006), and decreased BMP-2 expression (HR 2.348, p = 0.011) as significant predictive determinants of BCR (Table 4). GS (7, HR 2.836, p  =  0.022; ≥8, HR 3.506, p = 0.048) and decreased BMP-2 expression (HR 2.007, p = 0.047) remained significant predictors of BCR in multivariate analysis. Patients at stage ≥T3 in RP showed a 5-year BCR-free survival rate of 47.1%, whereas patients at other stages had a rate of 70.4% (p = 0.004; Fig. 2). In addition, the decreased BMP-2 expression group had lower BCR-free survival than the normal BMP-2 expression group according to Kaplan-Meier analysis (5-year BCR-free survival, 52.9% vs. 74.3%, p = 0.009). The 5-year BCR-free survival rates were 85.4% in the GS 6 group, 61.7% in the GS 7 group, and 29.2% in the group with GS ≥ 8 tumours (p < 0.05).

**Table 3:** Association of decreased BMP-2 expression with clinicopathological characteristics in 90 patients treated with radical prostatectomy for prostate cancer.

Variable	BMP-2 status		p-value
	Normal group (High and moderate expression) (N = 58)	Decreased group (Absent and Weak expression) (N = 32)	
Mean age, years	62.50 ± 5.98	62.94 ± 6.84	0.753
Mean PSA ± S.D	9.46 ± 6.85	8.41 ± 3.72	0.423
BMP expression (%)	44.83 ± 11.87	17.17 ± 0.82	0.001
Pathological tumour stage, n (%)			0.102
≤pT2c	51 (87.9%)	24 (75.0%)	
>pT2c	7 (12.1%)	8 (25.0%)	
Gleason score, n (%)			0.037
6	21 (36.2%)	7 (21.9%)	
7	35 (60.3%)	19 (59.4%)	
≥8	2 (3.4%)	6 (18.8%)	
Biochemical recurrence, n (%)	18 (31.0%)	18 (56.3%)	0.018
Progression to CRPC, n (%)	1 (1.7%)	3 (9.4%)	0.127

PSA = prostate specific antigen; BMP = bone morphogenetic proteins; S.D = standard deviation; CRPC = castration resistance prostate cancer.

**Table 4:** Results of univariate and multivariate Cox-proportional hazard analysis of clinico-pathological parameters and BMP-2 expression were differentially expressed with regard to the biochemical recurrence-free interval after radical prostatectomy.

Variable	Univariate		Multivariate	
	HR (95% CI)	p value	HR (95% CI)	p value
Age	1.016 (0.963–1.072)	0.566	N/A	N/A
Initial PSA	1.000 (0.943–1.059)	0.987	N/A	N/A
Immediate hormone treatment	0.462 (0.206–1.034)	0.060	N/A	N/A
GS				
6	Ref	Ref	Ref	Ref
7	3.000 (1.229–7.322)	0.016	2.836 (1.159–6.936)	0.022
≥8	5.081 (1.541–16.757)	0.008	3.506 (1.013–12.131)	0.048
T stage (>T2c)	2.718 (1.335–5.534)	0.006	1.795 (0.850–3.789)	0.125
PSM	2.032 (0.717–5.763)	0.182	N/A	N/A
Decreased BMP-2	2.348 (1.218–4.527)	0.011	2.007 (1.008–3.997)	0.047

BMP = bone morphogenetic proteins; CI = confidence interval; HR = hazard ratio; PSA = prostate-specific antigen; N/A = not applicable; PSM = positive surgical margin; GS = Gleason’s score.

## Discussion

Although the incidence of PCa has continued to increase, patient mortality has decreased remarkably. This phenomenon is partially due to early PCa detection, resulting from the widespread implementation of PSA screening, as well as significant advances in PCa therapeutics^19^. However, the utility of PSA as a prognostic factor in progressive PCa, unlike early-stage PCa, remains controversial. Semi-quantitative methods to evaluate PSA mRNA expression in circulating cells (using reverse transcriptase/polymerase chain reaction [PCR]) and various PSA constructs are among the post-treatment parameters most likely to be of prognostic significance. Although these laboratory observations are likely to be clinically relevant, assays used to evaluate separate drug effects on PSA secretion still require careful validation^20^.

Components of the TGF-β signalling pathway have been implicated in PCa regulation, with either tumour suppressor or tumour promoter activities being attributed to the pathway. More specifically, TGF-β signalling exhibits growth inhibitory effects in the early stages of PCa and promotes malignancy in later stages. Disruption of TGF-β signalling is referred to as a metastasis promoter^21^. BMPs, a subgroup of the TGF-β superfamily, were first described as factors inducing bone and cartilage formation; however, the BMP pathway was subsequently shown to be involved in cellular differentiation, organogenesis, chemotaxis, and cellular proliferation^22^. Among the BMPs, BMP-2 is known for its potent activities in inducing the entire cascade of cartilage and ectopic osteogenesis^23^. In addition to bone formation, BMP-2 plays an important role in cell differentiation, proliferation, morphogenesis, and apoptosis^24,25^. Moreover, BMP-2 is considered a putative tumour-suppressor gene in several cancer types.

A number of studies have examined BMP expression in normal and malignant human prostate tissues. Among the BMP family, only BMP-6 and BMP-7 have been shown to be associated with bone metastasis in PCa. Studies have found that osteoblastic bone lesions caused by PCa expressed BMP-6 and -7^26,27^.

However, the mechanism of BMP-2 expression as an indicator of oncological outcomes has not yet been elucidated. Spanjol and researchers reported high expression of BMP-2/4, -6, and -7 in PCa bone metastases^9^. Lai and colleagues also demonstrated that osteoblast-derived BMP-2 activated β1 integrin and β3 integrin and contributed to PCa cell migration^28^. In addition, Lao and investigators demonstrated that BMP-2 could stimulate PCa cell proliferation, tumour growth, and bone metastasis in an animal model^29^. The findings of these previous studies suggest that BMP-2 expression has potential prognostic value for cancer progression or survival in PCa patients. However, most previous studies examined metastatic PCa or were performed in animal models.

Some prior research suggested that BMP-2 expression in neoplastic prostate tissue is associated with PCa. For example, Doak and researchers reported that approximately half of the prostate tumours in their study displayed increased copy numbers of the BMP-2, BMP-5, and BMP-7 gene loci, which may account for their abnormal gene expression patterns in neoplastic prostate tissue^30^. However, the authors did not attempt to correlate the gene abnormalities with GS, positive resection margins, or PSA levels owing to the small study population (n = 12) analysed. In contrast, Horvath and colleagues demonstrated that decreased BMP-2 expression was associated with PCa progression, and its loss was associated with progression to a more aggressive phenotype^31^. The authors demonstrated that negative BMP-2 expression was associated with relapse-free survival time in Kaplan-Meier survival analysis and that loss of BMP-2 expression was correlated with increasing GS.

It was notable in the present analysis that among patients with PCa, decreased BMP-2 expression, rather than normal expression, was associated with BCR. In addition, BMP-2 expression was associated with the proportion of BCR and high GS in our cohort. As mentioned above, Horvath and colleagues also reported that loss of BMP-2 expression was correlated with increasing GS, similar to our results. However, they did not demonstrate a prognostic role for BMP-2 expression in the multivariate analysis.

Two other clinicopathologic factors, T stage and GS, were also confirmed as independent factors for predicting post-RP BCR. Many large centre studies have previously shown a similar result. Mani *et al*. presented in their large study that the strongest predictors of post-RP BCR were GS (over 8) and pathologic stage (>T3)^32^. In a large multicentre long-term follow-up study, among the 1061 patients with pathologic GS (>8), 80% who underwent RP will have experienced BCR by 15 years^33^. However, although many previous studies indicated that PSM could contribute to BCR, the role of PSMs in the development of BCR in patient subgroups with pT2-3a tumour stages remains controversial^34^. The reason that PSM was not significant in this study may be either because of the above results or because of the relatively small sample size of our study.

Although this retrospective study included a relatively small sample, the contributions of our study are as follows: to the best of our knowledge, this study is the first to report HRs of cumulative survival rates for decreased BMP-2 expression with its first revelation as an independent marker in a subsequent multivariate analysis. In addition, this was a relatively long-term follow-up study (over eight years in duration) performed to evaluate the prognostic value of BMP-2 with a focus on BCR in patients with PCa, which may assist clinicians in planning subsequent treatment following radical surgery. Furthermore, the other established prognostic factors, T stage and GS, were also analysed as prognostic factors for BCR-free survival in our study cohort. These results showed that our study cohort was representative of the general population.

There were some limitations to our study. As mentioned above, the study has a retrospective design and a relatively small study cohort from a single centre; moreover, some data were censored for some of the variables. In addition, we could not generate curves for progression-free survival (PFS) and overall survival, because patient progression to CRPC and death due to PCa were rare in this cohort. Nevertheless, to the best of our knowledge, this study constitutes the largest study sample to date for evaluation of the prognostic role of BMP-2 in PCa. If a longer study with a larger sample size could be conducted, PFS could be more clearly determined. Moreover, a more meaningful analysis could have been made based on the time to overall survival as well as PFS. In addition, the mechanism of BMP-2 on PCa is not completely understood. One possible explanation, suggested by Horvath *et al*., is that downregulation of BMP-2 signalling is related to control of cell proliferation^31^. However, there have been no studies to examine disruption of the BMP pathway in PCa. In addition, it is not clear whether this disruption is a primary or secondary phenomenon in prostate carcinogenesis. Further study is needed to more precisely define the mechanism of BMP pathway disruption in the progression of PCa and how this interacts with TGF-β signalling. Finally, we quantified BMP-2 expression by calculating the proportion of low-intensity area to total-image area. Previous researchers, including Asano and colleagues, have reported the current method^18^. However, the method used in this study has potential bias, which involves subjective factors. The BMP-2 level could have been quantified more accurately with western blotting or real-time PCR analysis. Recently, Yang and investigators used RNA analysis and real-time PCR to quantify BMP-2 expression. However, they did not conduct an analysis to associate BMP-2 levels with PCa prognosis^35^.

## Conclusion

Decreased BMP-2 expression in PCa tissue was correlated with a poor GS. In addition, it was also correlated with the important prognostic factors of BCR-free survival in patients with PCa. These data suggest that decreased BMP-2 expression in PCa tissue is related to progression to a more aggressive phenotype.
