# Author Correction: Decreased expression of bone morphogenetic protein-2 is correlated with biochemical recurrence in prostate cancer: Immunohistochemical analysis

**DOI:** 10.1038/s41598-018-29834-4

**Published:** 2018-07-27

**Authors:** Bum Sik Tae, Seok Cho, Hyun Cheol Kim, Cheol Hwan Kim, Seok Ho Kang, Jeong Gu Lee, Je Jong Kim, Hong Seok Park, Jun Cheon, Mi Mi Oh, Sung Gu Kang

**Affiliations:** 1 Department of Urology, Korea University College of Medicine, Seoul, Korea; 2 Department of Urology, Ilsan Paik Hospital, Inje University College of Medicine, Goyang, Korea; 3 Department of Pathology, Hallym University Dongtan Sacred Heart Hospital, Hwaseong, Republic of Korea; 4 Department of Pathology, Korea University College of Medicine, Seoul, Korea

Correction to: *Scientific Reports* 10.1038/s41598-018-28566-9, published online 16 July 2018

This Article contains typographical errors in the Acknowledgements section.

“This study was supported by a research grant (No. K1220301) from Korea University College of Medicine (Seoul, Korea).”

should read:

“This study was supported by a research grant (No. K1517861) from Korea University College of Medicine (Seoul, Korea).”

